# Serious infection risk of tofacitinib compared to biologics in patients with rheumatoid arthritis treated in routine clinical care

**DOI:** 10.1038/s41598-023-44841-w

**Published:** 2023-10-18

**Authors:** Myriam Riek, Almut Scherer, Burkhard Möller, Adrian Ciurea, Ines von Mühlenen, Cem Gabay, Diego Kyburz, Laure Brulhart, Johannes von Kempis, Ruediger B. Mueller, Paul Hasler, Tanja Strahm, Sabine von Känel, Pascal Zufferey, Jean Dudler, Axel Finckh

**Affiliations:** 1 SCQM Foundation, Aargauerstrasse 250, 8048 Zurich, Switzerland; 2 Inselspital und Universitätsspital Bern, Bern, Switzerland; 3 University Hospital Zurich, University of Zurich, Zurich, Switzerland; 4 Rheuma Basel, Private Practice, Basel, Switzerland; 5 Department of Internal Medicine, Rheumatology Division, University Hospitals Geneva, Geneva, Switzerland; 6 Department of Rheumatology, University Hospital Basel, Basel, Switzerland; 7 Rheumatology, Réseau Hospitalier Neuchâtelois, La Chaux-de-Fonds, Switzerland; 8 Division of Rheumatology and Immunology, Kantonsspital St.Gallen, St.Gallen, Switzerland; 9 Rheumazentrum Ostschweiz, Private Practice, St.Gallen, Switzerland; 10 University Medical Department, Division of Rheumatology, University of Basel Medical Faculty, Kantonsspital Aarau, Aarau, Switzerland; 11 Centres Hospitaliers Universitaires Vaudois, Lausanne, Switzerland; 12 Rhumatologie, HFR Fribourg, Hopital Cantonal, Fribourg, Switzerland

**Keywords:** Rheumatology, Rheumatic diseases, Drug safety

## Abstract

Recently, serious infections related to the use of tofacitinib (TOF) for treatment of rheumatoid arthritis (RA) have raised considerable interest. This study aimed to compare the risk for serious infections in patients with RA upon receiving TOF versus biologic disease-modifying antirheumatic drugs (bDMARDs) by age at treatment initiation. We identified adult RA patients exposed to TOF or bDMARDs using data collected by the Swiss registry for inflammatory rheumatic diseases (SCQM) from 2015 to 2018. The event of interest was the first non-fatal serious infection (SI) during drug exposure. Missing or incomplete SI dates were imputed as either the lower (left) or upper (right) limit of the known occurrence interval. The ratio of SI hazards (HR) of TOF versus bDMARDs was estimated as a function of age using covariate-adjusted Cox regression applied to each type of imputed time-to-SI. A total of 1687 patients provided time at risk for a first SI during study participation and drug exposure for 2238 different treatment courses, 345 for TOF and 1893 for bDMARDs. We identified 44 (left imputation) or 43 (right imputation), respectively, first SIs (12/12 on TOF versus 32/31 on bDMARDs). Left and right imputation produced similar results. For patients aged ≥ 69 years, the treatment HR started to be increased (lower limit of 95% confidence intervals (LLCIs) > 1). By the age of 76, the difference between TOF and bDMARDs started to be clinically relevant (LLCIs > 1.25). For patients aged < 65 years, the data were insufficient to draw conclusions. Our results suggest that we should expect an increased risk for SIs in older patients treated with TOF compared to bDMARDs supporting a cautious use of TOF in these patients.

## Introduction

Rheumatoid arthritis (RA) is a chronic, systemic autoimmune disease characterized by inflammation, persistent synovitis, and eventual joint destruction. If left untreated, RA can lead to increased morbidity and mortality^1,2^. Biologic and new targeted synthetic disease-modifying antirheumatic drugs (b/tsDMARDs) have dramatically improved RA care with unprecedented effectiveness in both easing symptoms and preventing joint damage^3^.

In August 2013, a novel synthetic DMARD, tofacitinib (TOF), that inhibits the Janus kinase family (JAK) was made available in the market^4^. Since then it has rapidly been adopted and widely used by the rheumatology community in Switzerland due to its effectiveness and ease of use^5^. The 2020 updated EULAR guidelines recommend JAK inhibitors, including TOF, as the second line of treatment alongside TNF inhibitors and non-TNF biologics in patients with moderate or high disease activity refractory to monotherapy with methotrexate^4,6^. However, rheumatologists and their RA patients need to choose between several licensed DMARDs without reliable predictors of individual responses to DMARDs^7^. Therefore, both the effectiveness and safety of the available options need to be considered when making a decision.

The risk of infections, including various bacterial and viral infections, as well as opportunistic infections, are of particular concern in patients with RA. Studies suggest that the disease process itself and the immunosuppressive properties of RA treatments contribute to increased infection risk^8^. However, the relative risks and types of serious infection may vary among DMARDs because of their different modes of action^9–11^.

The available literature on the comparative serious infection risk of TOF until recently was primarily based on observational data and was largely inconclusive^12–15^. The largest and most informative study published was based on data from US public and private health insurance programs^13^. This US study found indications for possible clinically relevant increased risks of serious infections for TOF compared to several bDMARDs while a study based on the US CORRONA RA registry comparing TOF to bDMARDs as a group could not exclude clinically relevant risk differences in either direction^14^. In 2019, interim results from an ongoing, open-label, randomized, post-authorization safety study (Study A3921133; NCT02092467) in RA patients aged 50 years or older and who were at an increased risk for cardiovascular adverse events suggested that TOF increases the risk of serious and fatal infections compared to the TNF inhibitors adalimumab and etanercept in the subset of patients aged 65 years or older^16,17^. Thereupon, the European Medicines Agency (EMA) recommended that “patients older than 65 years of age should be treated with TOF only when there is no alternative treatment”. On the other hand, the study based on US health insurance programs did not find a clear indication for such an increased risk in the Medicare database, a federally funded program providing health-care coverage for nearly all legal residents of the USA aged 65 years or older^13^. In the meantime, Study A3921133, also known as ORAL Surveillance, was published in 2022 and confirmed the interim results published in 2019^18,19^. We believe, however, that more data are needed to examine the risk of serious infections from TOF compared to bDMARDs in patients with RA or other indications and, particularly, how it changes with age.

In this study based on data from the Swiss registry for inflammatory rheumatic diseases (SCQM), we assessed the comparative risk for serious infections under TOF versus bDMARDs in RA patients as a function of age^20^.

## Methods

### Study design and study period

This observational cohort study is based on data collected by the SCQM registry from patients with RA in Switzerland^20^. The study provided additional financial or personnel support for data collection by rheumatologists during the study period from September 1 2015 to December 31 2018 to a subset of 23 institutions regularly participating in SCQM. Participating institutions were instructed to enroll and follow-up patients in SCQM as usual and collect and report data on their safety in a manner independent of the patient's treatment status. The 23 institutions, identified in Supplementary Methods, included all Swiss university hospitals, some tertiary care hospitals, and several private practices. Patients of interest were then retrospectively selected into this study based on SCQM’s database snapshot from July 1^st^ 2019 if they had been followed up by the institutions during the study period. For each enrolled patient the follow-up in the study ended at the last visit recorded until the end of the study.

### Patient population

Adult (18 years of age or older) RA patients were the population of interest. Other than that no inclusion or exclusion criteria were applied.

### Study outcome

The outcome of interest was the first non-fatal SI during drug exposure. Reported adverse events labeled as “infection” or “infestation” that were life-threatening, required hospitalization or prolongation of a hospitalization, or caused permanent disability or damage were identified as events of interest^21^. Fatal SIs were not observable during study participation, since a patient’s follow-up ended on the date of the last visit recorded by the institution before December 31 2018 inclusive.

### Exposures of interest

TOF and all bDMARDs approved for use in RA (comparator drug group) were of interest.

A patient was considered to be at a drug-related risk for SIs while actively exposed. The exposure period included the washout phase (the maximum of one day, one standard dosing interval, and twice the half-life) following the last administration. At any given time point, a patient’s drug-related SI risk was assumed to be determined by the drugs the patient was currently exposed to and the respective durations of continuous exposure at that point. I.e., treatments exposed to in the past were assumed to not affect a patient’s current risk, which was, hence, not considered different between patients with different drug experiences at the start of a new treatment. Any treatment course (TC) with a drug of interest fulfilling the following criteria was therefore considered for analysis: started after diagnosis and at the age of 18 years or older, was ongoing during study participation, had no concomitant exposure to other b/tsDMARDs, and the patient was at risk for a first SI (i.e., the patient had not experienced a SI since beginning of the TC). These criteria may not have applied to the entire TC, in which case only the period/s during which these criteria were fulfilled was/were considered. Gapless switches (i.e., switches within the washout period) between different brands of the same substance were considered as part of the same TC.

### Other variables of interest

The following variables were determined at study entry or the dates of treatment initiation: age, sex, seropositivity (as rheumatoid factor positivity or presence of CCP antibodies), disease duration from the date of diagnosis, DAS28-ESR, DAS28-CRP, time since the start of first b/tsDMARD therapy, current b/tsDMARD status (“naive”, “break”, “ongoing”, or “to be started”), past or current b/tsDMARD use (both number and identity of distinct, so far experienced b/tsDMARDs), previous exposure to prescribed b/tsDMARD, concomitant use of conventional synthetic (cs)DMARDs, concomitant use of oral glucocorticosteroids (GCS), known history of serious infections, and, in case of TOF, the dosing regimen. The variables assessed at study entry were used to describe the patients enrolled whereas those assessed at treatment starts were almost all used as covariates in the model. For a list of variables selected as covariates see the section on comparative risk later.

### Imputation of missing infection dates

A complete date (known year, month, and day) of occurrence was missing for some SIs. Based on the available date information and the time point of notification to SCQM, we derived an interval during which the event must have occurred. The left boundary or lower limit of that interval was the latest possible date for which we could assure that the event must have occurred thereafter, whereas the right boundary or upper limit was the earliest possible date for which we could assure that the event must have occurred previously. Depending on the available date information, the left boundary was set to either the first day of the month, the first day of the year, or, ultimately, the day of birth; the right boundary was set to either the last day of the month, the last day of the year, or the day the information was captured in the SCQM database. In cases where the event date was known exactly, the left and right boundary coincided. Examples are provided in Supplementary Table S1. Analyses were performed with the left boundary as left imputed date as well as with the right boundary as right imputed date.

### Statistical methods and considerations

Patient characteristics assessed at study entry and the start of the TC are summarized descriptively. Information on the number of observed SIs during study participation and the frequency and nature of inaccurately known dates is provided.

#### Comparative risk

To analyze the risk of SIs, we applied time-to-event methods with the origin of time set at start of the TC. In many cases, a patient was not observed from the start of treatment but from a later time point only. Either because the treatment had started before entry into the study or, alternatively, because the patient was initially exposed to more than one drug and single drug exposure (to either TOF or a single bDMARD) started only later. If, at this point, the patient was still at risk for a first SI they were included into the set of patients at risk as of that time point after start of treatment (delayed entry into the risk set or left truncation). Allowing for delayed entry, which preserves the timing with respect to the origin of time, enabled us to include (many) more TCs in the analysis. For a given TC, patients stayed in the risk set until occurrence of the first SI, end of exposure, start of another b/tsDMARD, or end of follow-up, whichever came first, with one of the latter three as first event resulting in a (right-)censored observation.

To assess the hazard ratio (HR) of TOF versus bDMARDs for a first SI as a function of age, we applied a Cox proportional hazards regression with covariates treatment and age and the treatment-age interaction together with a cluster term for patients (to account in a “generalized estimating equation”-like manner for the possible intra-patient correlation arising from contributing more than one TC). In addition to treatment and age, the following other covariates assessed at the start of each TC were considered in the model in order to reduce the possibility of confounding (based on conceptual considerations) or to adjust for important demographic or risk factors: sex, seropositivity, disease duration, concomitant csDMARD use, concomitant GCS use, and known history of serious infections. For continuos covariates (i.e., sex and disease duration) a linear relationship with the log hazard was assumed. For categorical covariates, we required at least five events per category for both date imputation types for the covariate to be considered in the model. For categorical, non-binary covariates, merging of categories was considered. The relevance of the interaction was evaluated based on the estimated treatment HR and the point-wise two-sided 95% confidence interval (CI) as a function of age and not primarily based on the p-value.

The analysis was performed twice, once based on left and once based on right imputed dates using a complete-case approach. The comparative risk was to be assessed if at least ten SIs were observed in total for both date imputations^22^.

Estimates of the first-year-incidence for first SI per 100 patients (i.e., assuming continuous observation of 100 patients at risk throughout the first year of treatment) by treatment group and age were derived as 100 times the respective estimated cumulative hazards for a first SI over the first year of treatment from the Cox proportional hazards regression.

Further details concerning data preparation and analysis (e.g., rationales for some of our choices like the selection of covariates and the use of Cox regression, covariate adjustment, or a complete-case approach) are provided in the Supplementary Methods.

#### Software

The data was prepared, analyzed, and reported using R (version 4.1.0) embedded in RStudio (version 1.4.1717)^23,24^. For the Cox proportional hazards regression we used the function coxph from the R package survival (version 3.2-11)^25^.

### Ethics approval and consent to participate

The SCQM registry has been approved by a national review board and all patients have given informed consent to their participation in SCQM.

Retrospective use of the data for this study was approved by the Ethical Committee of the canton of Vaud (CER-VD 2017-00619) on 15th of June 2017. The study was performed in accordance with relevant guidelines and regulations.

## Results

**Figure 1:**
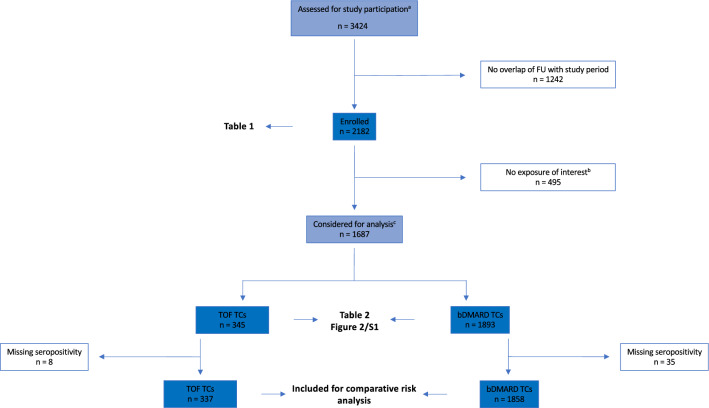
Patient flow chart. ^a^Patients ever followed up in SCQM as adult (18 + years) RA patients by participating institutions. ^b^No exposure to single biologic (b) or targeted synthetic (ts) disease-modifying antirheumtic drugs (DMARDs) during study participation that started after diagnosis, after 18th birthday, and during which the patient was at risk for a first non-fatal serious infection (SI). ^c^Patients who provided at least one day of at risk for a first SI from drugs of interest. *FU* follow-up, *TOF* tofacitinib, *TCs* treatment courses.

Out of the 3424 patients ever followed up in SCQM as adult RA patients by participating institutions, 2182 could be enrolled in the study (75% of which right at the start of the study). Table 1 summarizes the patient characteristics at study entry. Of the 2182 patients enrolled, 1687 contributed information on the risk for a first SI from 2238 TCs for a total duration of 3146.6 person-years (right date imputation). Of the 2238 TCs, 345 (from 327 patients) were with TOF and 1893 (from 1526 patients) with bDMARDs approved for RA. Further information regarding contribution of TCs by patients is provided in Supplementary Table S2. Table 2 summarizes the patient and treatment characteristics at the start of the TCs. A flowchart of patient enrollment and data availability is provided in Fig. 1.

**Table 1:** Summary of patient characteristics at study entry (n = 2182).

Variable	Number of patients	Statistic
Age (years), median (IQR^a^)	2182	60 (50–69)
≥ 50, n (%)		1686 (77.3)
≥ 65, n (%)		817 (37.4)
Female sex, n (%)	2182	1622 (74.3)
Seropositivity^b^, n (%)	2136	1580 (74.0)
Disease duration^c^ (years), median (IQR^a^)	2182	8 (4–16)
DAS28-ESR^d^, median (IQR^a^)	840	2.9 (2.0–4.0)
DAS28-CRP^d^, median (IQR^a^)	872	2.7 (1.8–3.6)
Current b/tsDMARD^e^ status, n (%)	2182	
Naive		492 (22.6)
Break		239 (10.9)
Ongoing^f^		1399 (64.1)
To be started^f^		52 (2.4)
b/tsDMARD experience^g^ (years), median (IQR^a^)	1655	6 (2–9)
Number of past or current b/tsDMARDs^h^, n (%)	2182	
0		527 (24.1)
1		733 (33.6)
2 or more		922 (42.2)
Identity of past or current b/tsDMARDs^h^, n (%)	1655	
Tofacitinib		192 (11.6)
Abatacept		445 (26.9)
Adalimumab		676 (40.9)
Anakinra		2 (0.1)
Certolizumab		112 (6.8)
Etanercept		552 (33.4)
Golimumab		224 (13.5)
Infliximab		409 (24.7)
Rituximab		459 (27.7)
Sarilumab		0 (0)
Tocilizumab		436 (26.3)
Off-label b/tsDMARDs		1 (0.1)
Known history of SI^i^, n (%)	2181	46 (2.1)

^a^*IQR* interquartile range.

^b^Rheumatoid factor positivity or presence of CCP antibodies.

^c^From date of diagnosis.

^d^Closest measurement within 90 days prior to study entry.

^e^b/tsDMARD: biologic (b)/targeted synthetic (ts) DMARD.

^f^Ongoing: considering the washout phase as well, to be started: a fresh start, i.e., no residual b/tsDMARD exposure.

^g^From start of very first b/tsDMARD.

^h^Distinct so far experienced b/tsDMARDs.

^i^SI: non-fatal serious infection.

**Table 2:** Summary of patient and treatment characteristics at the start of treatment courses by treatment.

Variable	TOF^a^ (n = 345 TCs)	bDMARDs^a^ (n = 1893 TCs)
Age (years), median (IQR^b^)	59 (50–69)	57 (48–66)
≥ 50, n (%)	263 (76.2)	1333 (70.4)
≥ 65, n (%)	126 (36.5)	522 (27.6)
Female sex, n (%)	270 (78.3)	1413 (74.6)
Seropositivity^c^, n (%)	226 (67.0^d^)	1406 (75.7^d^)
Disease duration^e^ (years), median (IQR^b^)	8 (4–17)	6 (2–14)
Concomitant csDMARDs^f^,	175 (50.7)	1313 (69.4)
Concomitant oral GCS^g^	133 (38.5)	622 (32.9)
Known history of SI^h^, n (%)	16 (4.6)	47 (2.5)
Delayed entry into risk set, n (%)		
Start prior to study entry	141 (40.9)	1229 (64.9)
Initially overlapping exposure	63 (18.3)	169 (8.9)
Previous exposure to treatment, n (%)	25 (7.2)	265 (14.0)
Initial dosing regimen, n (%)		
5 mg BID^i^	273 (79.1)	
5 mg OD^i^	18 (5.2)	
10 mg BID^i^	11 (3.2)	
10 mg OD^i^	38 (11.0)	
Other	5 (1.4)	
Identity of bDMARD, n (%)		
Abatacept		314 (16.6)
Adalimumab		227 (12.0)
Anakinra		1 (0.0)
Certolizumab		105 (5.5)
Etanercept		227 (12.0)
Golimumab		174 (9.2)
Infliximab		139 (7.3)
Rituximab		364 (19.2)
Tocilizumab		342 (18.1)

Number of patients: 1687.

^a^*TOF* tofacitinib, *bDMARDs* biologic DMARDs.

^b^*IQR* interquartile range.

^c^Rheumatoid factor positivity or presence of CCP antibodies.

^d^Number of TCs with available information: 337 for TOF, 1858 for bDMARDs.

^e^From date of diagnosis.

^f^*csDMARDs* conventional synthetic DMARDs.

^g^GCS: glucocorticosteroids.

^h^SI: non-fatal serious infection.

^i^*BID* twice daily, *OD* once daily.

**Figure 2:**
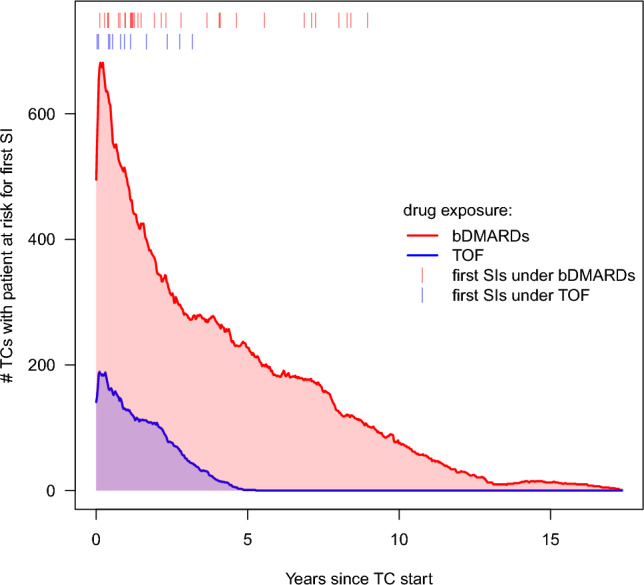
At-risk sets over time since treatment start by treatment. Shown are the number of treatment courses (TCs) with the patient at risk for a first non-fatal serious infection (SI) over time since treatment start by tofacitinib (TOF) and biologic disease-modifying antirheumtic drugs (bDMARDs). The total number of TCs with patients at observed risk was 2238. Due to delayed entry into the risk set the numbers do not decline monotonously. Vertical marks indicate the occurrence of a first SI. Inaccurate dates were right imputed. For left date imputation, the pattern looks very similar.

The number of TCs for which the patients were at risk for a first SI (sample size) as a function of time since exposure start by treatment is shown in Fig. 2. The areas under the curves graphically show not only the difference in number of TCs available by time since treatment start between the two treatment groups but also the fact that there is no information for TOF beyond five years after treatment start. The latter is due to TOF’s relatively recent entry into the market in August 2013. Supplementary Fig. S1 provides the same information additionally split by whether or not people were elderly (aged 65 years or older) at treatment start. For 636 of the 2238 TCs, patients were observed since the start of treatment. The patients responsible for the rest of the TCs (1602) made a delayed entry into the risk set, i.e., the TC started before the patient’s entry into the study or its initial period was not considered due to overlapping drug exposures.

In total, 67 SIs were identified whose occurrence overlapped with the 2182 patients’ study participation, all but two of which (still considered life-threatening) led to hospitalization. Fourteen (21%) of the identified SIs could not be dated accurately (11 were known by year and month, two by year only, for one date information was missing completely). Following left and right date imputation, 44 and 43 of the 67 events, respectively, were identified as first SIs on treatment. The one case with a missing date of occurrence (and a left imputed date set at date of birth) was not among these events as it would have happened, had it occurred during study participation, in a period of no b/tsDMARD exposure. We observed 12 first SIs during TOF and 32 during bDMARD exposure with left date imputation, and 12 and 31, respectively, with right date imputation. Of these, 10 for TOF and 13 for bDMARDs occurred in patients aged 65 years or older at treatment start.

**Figure 3:**
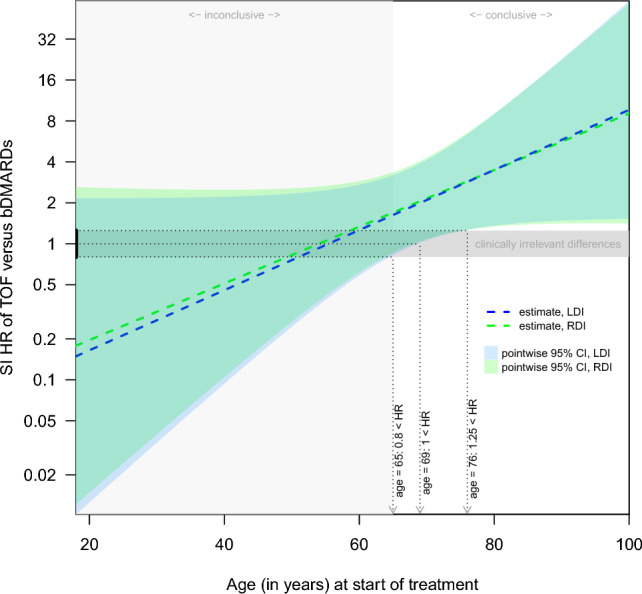
Estimated treatment hazard ratios and pointwise 95% confidence intervals for a first SI by age. Based on Cox proportional hazards regression with the following covariates: treatment [tofacitinib (TOF), biologic disease-modifying antirheumatic drugs (bDMARDs)], age, sex, seropositivity, disease duration, concomitant conventional synthetic DMARD use, concomitant glucocorticosteroid use, and known history of serious infections at start of treatment, and the interaction between treatment and age. Separate analyses were performed for left and right date imputation based on 2195 TCs (337 TOF, 1858 bDMARDs) from 1654 patients with information on seropositivity and 12/12 SIs for TOF and 32/31 for bDMARDs for left/right date imputation. The range for clinically irrelevant differences was set at 0.8 to 1.25. *HR* hazard ratio, *CI* confidence interval, *SI* non-fatal serious infection, *LDI/RDI* left/right date imputation, *TC* treatment course.

**Figure 4:**
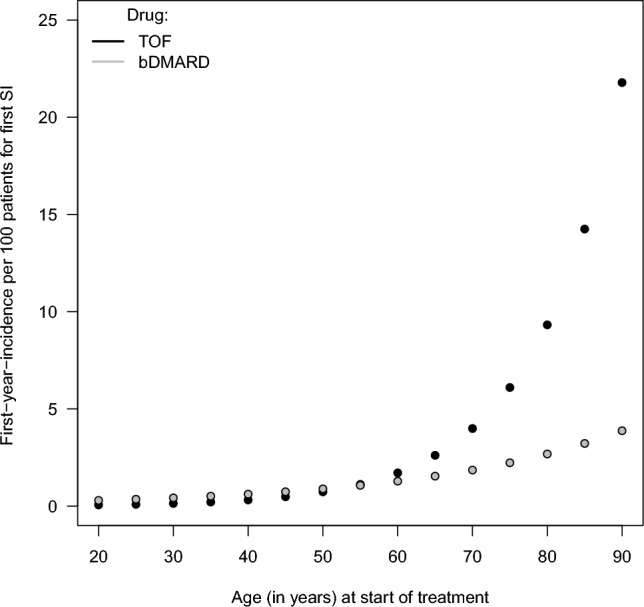
Estimated first-year-incidences per 100 patients for a first SI by treatment and age. Based on Cox proportional hazards regression as reported in Fig. 3. Inaccurate dates were right imputed. Estimates shown for a seropositive female patient diagnosed since ten years with concomitant conventional synthetic DMARD therapy without glucocorticosteroid use and no history of SIs. *SI* non-fatal serious infection, *TOF* tofacitinib, *(b)DMARDs* (biologic) disease-modifying antirheumatic drugs.

We assessed the age-dependent comparative risk of TOF versus bDMARDs by means of the HR as a function of age. As shown in Fig. 3, the hazard of a first SI due to TOF compared to bDMARDs was estimated to increase with increasing age by a factor of 1.05 (95% CI 1.00, 1.11) per year for left and right date imputation. However, the interaction with age was not statistically significant, meaning that our data cannot exclude an overall constant or even slightly decreasing comparative risk of TOF versus bDMARDs with age. Nevertheless, our results do suggest a comparable to a highly increased hazard for a first SI associated with TOF compared to bDMARDs at an age of 65 years, as judged from the 95% confidence intervals (CIs) of 0.83 to 3.19 and 0.86 to 3.35 for left and right date imputation, respectively. The results from both left and right date imputation also provide evidence for increased HRs for TOF compared to bDMARDs above the age of 69 years. Specifically, at 69 years the HRs were increased, at 1.99 (95% CI 1.02, 3.90) and 2.05 (95% CI 1.04, 4.05) for left and right date imputation, respectively. From the age of 76 onwards, the HR for TOF compared to bDMARDs was clinically relevantly increased, at 2.85 (95% CI 1.27, 6.38) and 2.87 (95% CI 1.27, 6.52) for left and right date imputation, respectively. For ages < 65, the data were insufficient to draw a conclusion one way or another. As estimates of absolute risk rates we provide the first-year-incidence per 100 patients by treatment and age derived from the Cox proportional hazards regression in Fig. 4 (right imputed dates) and Supplementary Fig. S2 (left imputed dates).

For comparison, we also ran the regression without the interaction term between treatment and age, assuming that the comparative risk is the same for all ages. The HRs (95% CIs) from these analyses were 1.80 (0.95, 3.43) for left and 1.88 (0.98, 3.60) for right date imputation. These values essentially coincide with those from the age-dependent analysis for an age of 67 years at start of treatment, which can be deduced from Fig. 3. Compared to the age-dependent analysis, this looks like an unreasonable extrapolation for younger ages.

The Cox proportional hazards regression was performed based on the 2195 TCs with complete covariate information and 44 and 43 first SIs, respectively, for left and right date imputation. I.e., we did not lose events due to missing covariate information. For details with respect to the fitted models see the Supplementary Results.

Based on data collected in 2019 and 2020 inclusive we established that 1396 of the 2182 enrolled patients had further visits since the end of the study on December 31 2018. The remaining 786 patients were lost to follow-up completely at their last visit before the study end (in SCQM structured follow-up of patients is bound to visits reported by rheumatologists). For these patients, SCQM has no information on the treatment status and occurrence of serious infections as of the time of loss to follow-up until the end of the study (or death of the patient). For five of the 786 patients we were informed about the discontinuation of their SCQM participation due to a fatal infection that happened after study participation and before the end of 2018. Further information regarding these five cases is provided in Supplementary Table S3.

## Discussion

In this observational cohort study, we evaluated the risk of first SIs under TOF compared to bDMARDs dependent on age at treatment start. Our results suggest that the risk of SIs under TOF compared to bDMARDs is increased as of an age of 69 as follows from the CIs (HR (95% CI): 1.99 (1.02, 3.90) for left and 2.05 (1.04, 4.05) for right date imputation). By the age of 76, the difference is suggested to reach clinical relevance (HR (95% CI): 2.85 (1.27, 6.38) for left and 2.87 (1.27, 6.52) for right date imputation). For patients younger than 65 years, our study remained inconclusive as neither clinically relevant increases nor decreases of the risk with TOF relative to bDMARDs could be ruled out, which was primarily due to a lack of such patients treated with TOF experiencing an SI.

Consistent with our findings, the results from the ORAL Surveillance trial in patients with cardiovascular risk factors suggested an increased risk of serious infections in elderly patients (aged 65 years or older) treated with TOF 10mg twice daily compared to TNF inhibitors^19^. The same study did, however, not find evidence for an increased risk with TOF administered at 5mg twice daily, the dosage taken by the majority of our patients. In contrast to ORAL Surveillance, more than 50% of our comparator TCs were not TNF inhibitors but rituximab, tocilizumab, and abatacept, suggesting that not only TNF inhibitors but also bDMARDs of other mechanisms of action could be associated with a lower risk for SIs in older patients compared to TOF. In contrast to our results, analyses of the Medicare database including elderly patients only did not reveal a clear indication for an increased risk of hospitalizations due to infections upon treatment with TOF compared to non-TNFi bDMARDs^12^. There was also no apparent difference in the comparative risk of TOF versus different bDMARDs from the Medicare database compared to two commercial national health-care claims databases with younger patient samples^13^. The study assessing data from phase II, III, and IIIb/IV TOF clinical trials and the CORRONA RA registry investigated the serious infection risk of TOF compared to adalimumab stratified by age^15^. Their data, too, did not provide evidence for an increased risk with TOF compared with adalimumab in patients 65 years or older. However, their confidence intervals were large. Nevertheless, in contrast to the US public and private health insurance programs study, they observed some indication for a larger comparative risk of TOF (at 10mg twice daily) in elderly patients compared to the non-elderly. Due to a lack of SIs observed in TOF-treated patients younger than 65 years of age, our study could not draw conclusions regarding the comparative risk for this age group and how it compares to the one in older patients. The role of age in TOF’s comparative infection risk remains thus ambiguous to date: It is unclear whether there is an age dependence and, if so, whether the risk of SIs with TOF compared to bDMARDs is enough increased in older compared to younger patients to justify a differential choice of treatments. In principle, it is plausible that different cellular pathways are differentially affected by aging processes leading to age-dependent treatment differences in both safety and effectiveness.

The results from the ORAL Surveillance trial led to a black box warning for the class of JAK inhibitors by several health authorities suggesting that the transferability of findings related to TOF to other JAK inhibitors was considered too substantial to be ignored. Studies with other JAK inhibitors like baricitinib or upadacitinib are definitely needed to assess whether the increased risk of SIs in older patients compared to available alternatives is a class effect or a drug effect related to TOF.

Our event of interest, non-fatal serious infections, deviates from the type of event considered in the above-cited studies. Two of the studies evaluated infections requiring hospitalization and all included fatal events. Almost all our events considered for the analyses were also labeled as having required hospitalization and, if not so, were considered as life-threatening. In these cases it is likely that hospitalization had occurred but was not recorded. Moreover, the comparative risk for non-fatal serious infections is also representative for the comparative risk for serious infections in general if the impact of treatments on the risks for fatal and non-fatal serious infections is the same or the risk for fatal serious infections is relatively small. On the other hand, differentiating between fatal and non-fatal serious infections can be of value on its own, especially if the impact of treatments differ. The latter possibility is supported by studies suggesting a beneficial effect of bDMARDs compared to csDMARDs on the risk of a fatal infection outcome, although an increased risk for experiencing a serious infection in the first place with bDMARDs compared to csDMARDs is generally assumed^9–11,26^. So, even though we were unable to assess the comparative risk for fatal serious infections, we believe that our assessment of non-fatal events provides valuable information.

Several studies reported the possibility of a dose dependence for infections under TOF^15,17,19^. In our study, the majority of patients were treated with the recommended 5mg of TOF twice daily or an equivalent or smaller total daily dose (Table 2) and we therefore did not account for different dosing regimens in our analyses.

Our study has several limitations. First, we faced uncertainty in the timing of some SIs. To accommodate this uncertainty, we imputed incomplete or missing dates and considered the values contained by either confidence interval (based on left and right imputed dates, respectively) as plausible for the true HR. Second, our study was not blinded, and the reporting of infection events may have been influenced by knowing a patient's treatment status despite instructions to the contrary. Third, the underreporting of events is a general issue for optional registries and likely to have occurred in this study despite the provision of financial or personnel support for data collection. Furthermore, we note that our comparison of TOF and bDMARDs was roughly based on data up to three years of treatment only. Thereafter, data for TOF were scarce or inexistent. Our findings do thus not apply to long-term treatment risk comparisons. Finally, our sample size was too small to allow conclusions regarding the age-dependent comparative risk of TOF versus bDMARDs; we were merely able to support findings from the literature for older patients.

Due to the lack of data on TOF safety in the general patient population at the time of market entry, we planned this study nested within the SCQM. The events of interest were serious infections, malignancies, and major adverse cardiovascular events. In this article, we report our findings for serious infections. We intend to report our findings for malignancies and major adverse cardiovascular events in a separate article. Our focus was on serious infections in general, and we did not analyze specific subgroups due to the small number of observed events.

The study’s observational nature means that we must exercise caution when interpreting the observed associations causally, as we cannot rule out residual confounding of the treatment effect. Nonetheless, in our view, this study addressed the problem of confounding adequately enough to provide sufficiently reliable information on the comparative risk of serious infections with b/tsDMARDs as faced by rheumatologists and RA patients when deciding on treatments.

## Conclusions

This study suggests that an increased risk for SIs is to be expected in older patients (aged ≥ 69 years) under treatment with TOF compared to bDMARDs. It therefore represents an independent source of support for current recommendations to use TOF cautiously in patients aged 65 years or older. Further research is needed to assess whether the comparative risk is age dependent and, if so, whether it is relevantly increased in older as opposed to younger patients.

### Supplementary Information

Supplementary Information.

## Data Availability

The data underlying this article were provided by the SCQM Foundation by permission. Data may be shared on reasonable request to the corresponding author with permission of the SCQM Foundation.

## Abbreviations

BID: Twice daily dosing
CCP: Cyclic citrullinated peptide
CORRONA: Consortium of rheumatology researchers of North America
CI: Confidence interval
DAS28-CRP: Disease activity score 28 based on C-reactive protein
DAS28-ESR: Disease activity score 28 based on erythrocyte sedimentation rate
DMARD (b-, ts-, cs-): Disease modifying antirheumatic drug (biologic, targeted synthetic, conventional synthetic)
EMA: European Medicine Agency
FDA: Food and Drug Administration
FU: Follow-up
GCS: Glucocorticosteroids
HR: Hazard ratio
IQR: Interquartile range
JAK: Janus kinase family
LDI: Left date imputation
OD: Once daily dosing
RA: Rheumatoid arthritis
RDI: Right date imputation
SCQM: Swiss clinical quality management in rheumatic diseases
SI: (Non-fatal) serious infection
TC: Treatment course
TNF(i): Tumor necrosis factor (inhibitor)
TOF: Tofacitinib
