# Supplementary material for: Serious infection risk of tofacitinib compared to biologics in patients with rheumatoid arthritis treated in routine clinical care
Source: Sci Rep. 2023 Oct 18;13:17776. doi: 10.1038/s41598-023-44841-w (PMC10584888; doi:10.1038/s41598-023-44841-w)
Supplement: Supplementary file 1 — Supplementary Information. [file 41598_2023_44841_MOESM1_ESM.docx]

*Serious infection risk of tofacitinib compared to biologics in patients with rheumatoid arthritis treated in routine clinical care –* ***supplementary material***

Myriam Riek, Almut Scherer, Burkhard Möller, Adrian Ciurea, Ines von Mühlenen, Cem Gabay, Diego Kyburz, Laure Brulhart, Johannes von Kempis, Ruediger B Mueller, Paul Hasler, Tanja Strahm, Sabine von Känel, Pascal Zufferey, Jean Dudler, Axel Finckh

**METHODS**

**Participating institutions**

Cabinet Martin du Pan, Geneva; Inselspital - University Hospital Bern, Bern; Kantonsspital Aarau, Aarau ; Hôpital Cantonal, Fribourg, Fribourg; Kantonsspital St.Gallen, St.Gallen; OsteoRheuma Bern, Bern; Cabinet Buchs Nicolas, Geneva; Praxis Exer / von Mühlenen, Basel; Praxis Hüllstrung / Iseli, Liestal; Praxis Klöti, Luzern; Cabinet Stingelin Guerne, Versoix; Rheumazentrum Aarau, Aarau; Réseau hospitalier neuchâtelois, La Chaux-de-Fonds; University Hospital Basel, Basel; University Hospitals Geneva, Geneva; Centres Hospitaliers Universitaires Vaudois, Lausanne; University Hospital Zurich, Zurich; Medizinisches Zentrum Brugg, Brugg; Rheumatologie Oberaargau, Langenthal; Hirslanden Klinik Birshof, Münchenstein; Berner Rheumazentrum, Bern; Bethesda Spital, Basel; Praxis Martin, Liestal

**Date imputation**

**Supplementary Table S1: Examples of imputed event dates.**

| **Available date information** | **Left imputed date** | **Right imputed date** |
| --- | --- | --- |
| **Exact date:** |  |  |
| 2017-04-04 | 2017-04-04 | 2017-04-04 |
|  |  |  |
| **Year and month known:** |  |  |
| 2018-05-dd | 2018-05-01 | Minimum of 2018-05-31 and date of recording |
|  |  |  |
| **Year known:** |  |  |
| 2017-mm-dd | 2017-01-01 | Minimum of 2017-12-31 and date of recording |
|  |  |  |
| **Unknown:** | Date of birth | Date of recording |
|  |  |  |

**Statistical methods**

The event of interest was the first non-fatal serious infection (SI) occurring during a given course of continuous b/tsDMARD exposure. A major competing event in this setting was termination of exposure to the b/tsDMARD without a prior SI. As we assessed the effect of treatment on the hazard only and did not derive cumulative incidence estimates, we did not consider the competing events in the analyses.

***Rationales for method choices***

*Confounding of the treatment-outcome association*: The variables needed to prevent or lessen the possibility of confounding were selected based on conceptual considerations. Variables considered to be likely common causes of treatment assignment and outcome or thought to lie on the causal path from such a common cause to either treatment assignment or outcome were selected as covariates. Specifically, based on the chosen risk concept as explained in the manuscript, a covariate measuring the extent of previous exposure to b/tsDMARDs was not considered necessary from a confounding perspective.

*Covariate adjustment*: We chose to estimate a conditional instead of an average treatment effect because we were specifically interested in assessing the treatment-age interaction and therefore opted for the use of covariate adjustment.

*Cox proportional hazards regression*: The choice of a Cox proportional hazards regression as opposed to a Poisson regression was based on the fact that the former does not restrict the form of the hazard function to be constant, an assumption that in our perspective would not be justified. Moreover, the Cox regression needs to define an origin of time that should be a relevant and non-arbitrary point in time. For this study, the origin of time was set at the start of TCs, i.e., the point in time when the future risks and benefits of treatments are evaluated and compared.

*Complete-case approach*: We adopted a complete-case approach in the event of missing covariate information. A complete-case approach is unbiased in case missingness is independent of the outcome variable conditional on the model covariates.[1] Since we considered this condition to be likely met, we opted for the complete-case approach, at the possible expense of losing information.

**RESULTS**

**Contribution of treatment courses (TCs) by patients**

A total of 404 of the 1687 patients contributed more than one TC. In the following table we provide numbers for specific contribution patterns.

**Supplementary Table S2: Treatment course contribution patterns.**

| **Case** | **Number of patients** |
| --- | --- |
| Only several TOF TCs | 6 |
| Only several bDMARD TCs | 232 |
| bDMARD TCs following TOF initially^a^ | 47 |
| TOF TCs following bDMARD initially^a^ | 119 |

^a^: Does not exclude further TOF or bDMARDs, respectively.

TOF: tofacitinib, TC: treatment course, bDMARD: biologic disease-modifying antirheumatic drug.

**Supplementary Figure S1: At-risk sets over time since treatment start by treatment and elderly status**. Shown the number of treatment courses (TCs) with the patient at risk for a first non-fatal serious infection (SI) over time since start of treatment by treatment and elderly status at start of the TC. Elderly was defined as an age of 65 years or older. The total number of TCs with patients at observed risk was 2238. Due to delayed entry into the risk set the numbers do not decline monotonously. Vertical marks indicate the occurrence of a first SI. Inaccurate dates were right imputed. For left date imputation, the pattern looks highly similar. TOF: tofacitinib, bDMARDs: biologic disease-modifying antirheumatic drugs.

**Supplementary Figure S2: Estimated first-year-incidences per 100 patients for a first SI by treatment and age**. Based on Cox proportional hazards regression as reported in Figure 3. Inaccurate dates were left imputed. Estimates shown for a seropositive female patient diagnosed since ten years with concomitant conventional synthetic DMARD therapy without glucocorticosteroid use and no history of SIs. SI: non-fatal serious infection, TOF: tofacitinib, (b)DMARDs: (biologic) disease-modifying antirheumatic drugs.

**Cox model outputs**

R package: survival (version 3.2-11), function: coxph()

| **Summary of Cox PH regression fit based on left imputed dates for non-fatal serious infections (SIs):** | | | | | | | |
| --- | --- | --- | --- | --- | --- | --- | --- |
| **Time scale: time since start of treatment course (TC)** | | | | | | | |
| **Number of TCs = 2195, number of SIs = 44** | | | | | | | |
| **Comparator for drug exposure: bDMARDs** | | | | | | | |
|  |  |  |  |  |  |  |  |
|  | **Coefficient** | **SE (robust)** | **P-value^a^** | **HR^b^** | **Ratio of HRs^c^** | **LCL^d^** | **UCL^d^** |
| **With treatment-age interaction** |  |  |  |  |  |  |  |
|  |  |  |  |  |  |  |  |
| male vs female sex | 0.356967663 | 0.31461672 | 0.257 | 1.428990 |  | 0.7713062 | 2.647472 |
| +1 year in age at TC start for bDMARDs | 0.035271188 | 0.01523731 | 0.021 | 1.035901 |  | 1.0054213 | 1.067304 |
| seropositivity vs -negativity | 0.691591767 | 0.52673624 | 0.189 | 1.996892 |  | 0.7112066 | 5.606776 |
| +1 year disease duration at TC start | 0.004504987 | 0.01375179 | 0.743 | 1.004515 |  | 0.9778021 | 1.031958 |
| known vs unknown history of SIs at TC start | 0.624467002 | 0.47246022 | 0.186 | 1.867250 |  | 0.7396797 | 4.713695 |
| no concomitant vs concomitant csDMARDs at TC start | 0.565594086 | 0.32169440 | 0.079 | 1.760493 |  | 0.9371468 | 3.307205 |
| no concomitant vs concomitant GCS at TC start | 0.027174649 | 0.32269347 | 0.933 | 1.027547 |  | 0.5459145 | 1.934100 |
| TOF vs bDMARDs at age 60 yrs at TC start | 0.232004375 | 0.39129102 | 0.553 | 1.261125 |  | 0.5857209 | 2.715349 |
| difference of +1 year in age at TC start for TOF vs bDMARDs | 0.050879022 | 0.02681870 | 0.058 |  | 1.052196 | 0.9983168 | 1.108982 |
|  |  |  |  |  |  |  |  |
| **Without treatment-age interaction** |  |  |  |  |  |  |  |
|  |  |  |  |  |  |  |  |
| male vs female sex | 0.355984739 | 0.31616520 | 0.260 | 1.427586 |  | 0.7682134 | 2.652910 |
| +1 year in age at TC start | 0.046458266 | 0.01347592 | < 0.001 | 1.047554 |  | 1.0202482 | 1.075591 |
| seropositivity vs -negativity | 0.654727524 | 0.52622702 | 0.213 | 1.924618 |  | 0.6861504 | 5.398459 |
| +1 year disease duration at TC start | 0.005026958 | 0.01377458 | 0.715 | 1.005040 |  | 0.9782689 | 1.032543 |
| known vs unknown history of SIs at TC start | 0.640079559 | 0.46617763 | 0.170 | 1.896632 |  | 0.7606273 | 4.729270 |
| no concomitant vs concomitant csDMARDs at TC start | 0.581540830 | 0.32051741 | 0.070 | 1.788793 |  | 0.9544102 | 3.352624 |
| no concomitant vs concomitant GCS at TC start | 0.026439181 | 0.32094139 | 0.934 | 1.026792 |  | 0.5473897 | 1.926053 |
| TOF vs bDMARDs | 0.590194604 | 0.32765471 | 0.072 | 1.804340 |  | 0.9493319 | 3.429402 |

^a^ Wald test

^b^ HR: hazard ratio = exp(coefficient)

^c^ Ratio of treatment specific HR with respect to one year increase in age = exp(coefficient)

^d^ Lower (LCL) and upper (UCL) limit of two-sided 95% Wald-type confidence interval

| **Summary of Cox PH regression fit based on right imputed dates for non-fatal serious infections (SIs):** | | | | | | | |
| --- | --- | --- | --- | --- | --- | --- | --- |
| **Time scale: time since start of treatment course (TC)** | | | | | | | |
| **Number of TCs = 2195, number of SIs = 43** | | | | | | | |
| **Comparator for drug exposure: bDMARDs** | | | | | | | |
|  |  |  |  |  |  |  |  |
|  | **Coefficient** | **SE (robust)** | **P-value^a^** | **HR^b^** | **Ratio of HRs^c^** | **LCL^d^** | **UCL^d^** |
| **With treatment-age interaction** |  |  |  |  |  |  |  |
|  |  |  |  |  |  |  |  |
| male vs female sex | 0.410193946 | 0.31549287 | 0.194 | 1.507110 |  | 0.8120765 | 2.797003 |
| +1 year in age at TC start for bDMARDs | 0.036923900 | 0.01591266 | 0.020 | 1.037614 |  | 1.0057521 | 1.070485 |
| seropositivity vs -negativity | 0.667740092 | 0.52979150 | 0.208 | 1.949826 |  | 0.6902978 | 5.507508 |
| +1 year disease duration at TC start | 0.005572588 | 0.01372482 | 0.685 | 1.005588 |  | 0.9788983 | 1.033006 |
| known vs unknown history of SIs at TC start | 0.633374209 | 0.47569660 | 0.183 | 1.883957 |  | 0.7415787 | 4.786131 |
| no concomitant vs concomitant csDMARDs at TC start | 0.616245987 | 0.32556144 | 0.058 | 1.851963 |  | 0.9783941 | 3.505505 |
| no concomitant vs concomitant GCS at TC start | 0.101398028 | 0.32932566 | 0.758 | 1.106717 |  | 0.5803822 | 2.110372 |
| TOF vs bDMARDs at age 60 yrs at TC start | 0.288223999 | 0.39315646 | 0.463 | 1.334056 |  | 0.6173319 | 2.882899 |
| difference of +1 year in age at TC start for TOF vs bDMARDs | 0.047953935 | 0.02697326 | 0.075 |  | 1.049122 | 0.9950994 | 1.106078 |
|  |  |  |  |  |  |  |  |
| **Without treatment-age interaction** |  |  |  |  |  |  |  |
|  |  |  |  |  |  |  |  |
| male vs female sex | 0.410743146 | 0.31654703 | 0.194 | 1.507938 |  | 0.8108456 | 2.804328 |
| +1 year in age at TC start | 0.047818320 | 0.01395695 | < 0.001 | 1.048980 |  | 1.0206740 | 1.078071 |
| seropositivity vs -negativity | 0.629137040 | 0.52828335 | 0.234 | 1.875991 |  | 0.6661241 | 5.283313 |
| +1 year disease duration at TC start | 0.006145366 | 0.01373125 | 0.654 | 1.006164 |  | 0.9794468 | 1.033611 |
| known vs unknown history of SIs at TC start | 0.651492447 | 0.46783332 | 0.164 | 1.918402 |  | 0.7668654 | 4.799102 |
| no concomitant vs concomitant csDMARDs at TC start | 0.631186184 | 0.32474120 | 0.052 | 1.879839 |  | 0.9947191 | 3.552556 |
| no concomitant vs concomitant GCS at TC start | 0.101191459 | 0.32723177 | 0.757 | 1.106488 |  | 0.5826486 | 2.101295 |
| TOF vs bDMARDs | 0.630637184 | 0.33214994 | 0.058 | 1.878807 |  | 0.9798412 | 3.602540 |

^a^ Wald test

^b^ HR: hazard ratio = exp(coefficient)

^c^ Ratio of treatment specific HR with respect to one year increase in age = exp(coefficient)

^d^ Lower (LCL) and upper (UCL) limit of two-sided 95% Wald-type confidence interval

**Information on fatal infections after study participation**

Based on data collected in 2019 and 2020 inclusive, we established that 786 of the 2182 patients enrolled were definitively lost to follow-up in SCQM before the end of the study on December 31 2018. For these patients, SCQM has no information on the treatment status and the occurrence of serious infections from the time of loss to follow-up until the end of the study (or death of the patient). We were informed that discontinuation of SCQM participation due to a fatal infection had happened before December 31 2018 for five of the 786 patients. Since structured follow-up of patients in SCQM is bound to visits reported by the rheumatologist, the timing of the infection, the treatment status at time of infection as well as the absence of prior non-fatal serious infections could not be confirmed. For these five cases, information on the last known treatment status at loss to follow-up, and the time window for death in days from loss to follow-up are listed in Table S1.

**Supplementary Table S3 Information on fatal infections after study participation and before the end of the study.** b/tsDMARD: biologic / targeted synthetic disease-modifying antirheumatic drug, FU: follow-up, TOF: tofacitinib.

| **Case number** | **Ongoing b/tsDMARD at end of FU^a^** | **Occurrence of death**  **(window in days from end of FU)** |
| --- | --- | --- |
| 1 | TOF | (0, 383) |
| 2 | bDMARD | (438, 488) |
| 3 | bDMARD | (0, 487) |
| 4 | bDMARD | (0, 106) |
| 5 | none | (409, 516) |

^a^: End of follow-up in SCQM coincides with the end of study participation for all five patients

**REFERENCES**

1. White, I. R. & Carlin, J. B. Bias and efficiency of multiple imputation compared with complete-case analysis for missing covariate values. *Stat Med*. **29(28),** 2920-2931 (2010).
